# MicroRNAs in Colon Tissue of Pediatric Ulcerative Pancolitis Patients Allow Detection and Prognostic Stratification

**DOI:** 10.3390/jcm10061325

**Published:** 2021-03-23

**Authors:** Petr Jabandziev, Tatsuhiko Kakisaka, Julia Bohosova, Tereza Pinkasova, Lumir Kunovsky, Ondrej Slaby, Ajay Goel

**Affiliations:** 1 Department of Pediatrics, University Hospital Brno, Faculty of Medicine, Masaryk University, 613 00 Brno, Czech Republic; jabandziev.petr@fnbrno.cz (P.J.); pinkasova.tereza@fnbrno.cz (T.P.); 2 Central European Institute of Technology, Masaryk University, 625 00 Brno, Czech Republic; jul.bohosova@gmail.com (J.B.); on.slaby@gmail.com (O.S.); 3 Department of Molecular Diagnostics and Experimental Therapeutics, Beckman Research Institute of City of Hope Comprehensive Cancer Center, Duarte, CA 91010, USA; puripurikoyu@gmail.com; 4 Department of Gastroenterology and Internal Medicine, University Hospital Brno, Faculty of Medicine, Masaryk University, 625 00 Brno, Czech Republic; kunovsky.lumir@fnbrno.cz; 5 Department of Surgery, University Hospital Brno, Faculty of Medicine, Masaryk University, 625 00 Brno, Czech Republic; 6 Department of Biology, Faculty of Medicine, Masaryk University, 625 00 Brno, Czech Republic

**Keywords:** pediatrics, inflammatory bowel disease, ulcerative colitis, microrna, primary sclerosing cholangitis

## Abstract

Prevalence of inflammatory bowel disease has been on the rise in recent years, especially in pediatric populations. This study aimed to provide precise identification and stratification of pediatric patients with diagnosed ulcerative colitis (UC) according to the severity of their condition and the prediction for standard treatment according to the specific expression of candidate miRNAs. We enrolled consecutive, therapeutically naïve, pediatric UC patients with confirmed pancolitis. We examined formalin-fixed paraffin-embedded specimens of colonic tissue for the expression of 10 selected candidate miRNAs. We performed receiver operating characteristic curve analysis, using area under the curve and a logistic regression model to evaluate the diagnostic and predictive power of the miRNA panels. Sixty patients were included in the final analysis. As a control group, 18 children without macroscopic and microscopic signs of inflammatory bowel disease were examined. The combination of three candidate miRNAs (let-7i-5p, miR-223-3p and miR-4284) enabled accurate detection of pediatric UC patients and controls. A panel of four candidate miRNAs (miR-375-3p, miR-146a-5p, miR-223-3p and miR-200b-3p) was associated with severity of UC in pediatric patients and a combination of three miRNAs (miR-21-5p, miR-192-5p and miR-194-5p) was associated with early relapse of the disease. Nine patients out of the total were diagnosed with primary sclerosing cholangitis (PSC) simultaneously with ulcerative colitis. A panel of 6 candidate miRNAs (miR-142-3p, miR-146a-5p, miR-223-3p, let-7i-5p, miR-192-5p and miR-194-5p) identified those patients with PSC. Specific combinations of miRNAs are promising tools for potential use in precise disease identification and severity and prognostic stratification in pediatric patients with ulcerative pancolitis.

## 1. Introduction

Inflammatory bowel disease (IBD) is a chronic complex disorder of the digestive system caused by multiple factors, including genetics, epigenetics, gut microbiota, environmental factors and altered immune system, although the precise mechanisms underlying the pathogenesis of this disease remain unclear [1,2,3]. Owing to chronic inflammation, patients with ulcerative colitis (UC) and Crohn’s disease (CD) involving the colon have an increased risk for colon cancer. The risk increases with time from diagnosis and higher-risk groups include those younger at diagnosis, with pancolitis, or with associated primary sclerosing cholangitis (PSC) [4,5].

Although prevalence of the disease peaks in the fourth and fifth decades of life, a substantial number of patients have onset of IBD during early childhood [6,7]. Significant differences in clinical manifestation, possible pathophysiology and treatment between children and adults prevent extrapolation of knowledge from one group to the other [8]. Studies have shown that phenotypes of pediatric patients with IBD are more aggressive and with more damaging impact disrupting normal growth and overall development of the child [9]. Moreover, therapeutically naïve patients often show quite different responses to the treatment given [10,11]. Despite outstanding achievements in therapeutic interventions, there is a clear need for biomarkers enabling accurate identification, prognosis and prediction of response to therapies to facilitate a more individualized approach. In recent years, evidence has continued to evolve concerning non-coding RNAs (ncRNAs) and their roles as integral factors in key immune-related cellular pathways. Specific deregulation patterns of ncRNAs have been linked to the pathogenesis of adult and pediatric IBD [12]. Several microRNAs (miRNAs) and specific miRNA signatures have been identified in IBD-associated tissues. It has been shown that, among many other cellular processes, miRNAs play a significant role in intestinal immunity. Nevertheless, there exists only sparse information on ncRNA profiles and their diagnostic and prognostic potential in pediatric IBD patients [12]. In this article, we stratify therapeutically naïve pediatric patients with diagnosed UC according to the severity of their condition and the prediction for standard treatment according to the specific expression of 10 candidate miRNAs.

## 2. Materials and Methods

### 2.1. Study Design

Subjects were enrolled at the Department of Pediatrics, University Hospital Brno, Czech Republic. Eligible subjects consisted of children having been diagnosed with UC (only pancolitis) according to relevant guidelines [13,14] (clinical history, physical examination, laboratory results, serological testing, radiologic findings and endoscopic appearance with stepwise biopsy for review by clinical pathologists) in a period between 1 January 2012 and 31 December 2018. All patients underwent upper gastrointestinal endoscopy and ileocolonoscopy. All were 0–18 years of age at the time of diagnosis and were resident in the South Moravian Region, one of 14 regional administrative units in the Czech Republic [6]. Enrolled into the study were only unequivocal UC cases with pancolitis (with proven macroscopic and microscopic disease affection) as determined by the Paris classification (E4) [15]. Patients without indisputable diagnosis of UC according to the Porto criteria were excluded from further analyses [14]. Initial severity of the disease was evaluated by an experienced gastroenterologist using the pediatric ulcerative colitis activity index (PUCAI) score [16]. In our other analyses, we focused only on patients with mild (PUCAI 10–34) and moderate (PUCAI 35–64) ulcerative pancolitis. Patients with severe colitis (PUCAI > 65) were excluded from the study for a low number of patients (n = 3) and lack of representative material for an examination. We examined formalin-fixed paraffin-embedded specimens of colonic tissue (rectosigmoid colon).

We focused on precise identification of pediatric UC patients and, thereafter, on possible stratification for severity of disease according to specific patterns of candidate miRNAs’ expression in colonic tissue. We then undertook to create a candidate model for predicting early relapse of the disease. Patients received initial standardized treatment with mesalazine and/or corticosteroids guided by the PUCAI according to relevant national guidelines [17] and were evaluated for adherence to therapy by use of structured interview. We monitored whether after standard treatment a patient experienced an early relapse during the first year of treatment despite significant initial remission of the disease, defined as PUCAI < 10 during the first 3 months after diagnosis and fecal calprotectin <100 ug/L. Moreover, we compared patients with diagnosed PSC and patients without this diagnosis.

As a control group, we used children indicated for the endoscopic examination mostly due to chronic abdominal pain, but these patients did not show neither macroscopic nor microscopic signs of IBD and fecal calprotectin was normal. None of the patients were diagnosed with IBD one year after enrollment in the study.

The Institutional Ethical Committee approved the study at University Hospital Brno in accordance with the Declaration of Helsinki.

### 2.2. Candidate miRNAs Selection

A reasonable number of candidate miRNAs were selected based on a literature review published elsewhere [12]. These were only demonstrably dysregulated miRNAs in terms of downregulation and upregulation in pediatric patients: 5 upregulated (miR-21-5p, miR-142-3p, miR-146a-5p, miR-223-3p and let-7i-5p) and 5 downregulated (miR-192-5p, miR-194-5p, miR-200b-3p, miR-375-3p and miR-4284).

### 2.3. Nucleic Acid Isolation and miRNA Expression Analysis

Total RNA was isolated from formalin-fixed paraffin-embedded biopsy tissues from rectosigmoideum using ALL Prep DNA/RNA FFPE kit (Qiagen, Valencia, CA, USA). Synthesis of complementary DNA (cDNA) was conducted using miRCURY LNA RT Kit (Qiagen, Valencia, CA, USA). miRCURY LNA miRNA PCR Assays (primer mix) for the following miRNAs were used for quantitative real-time reverse transcription analysis (qRT-PCR): miR-16-5p, miR-21-5p, miR-142-3p, miR-146a-5p, miR-192-5p, miR-194-5p, miR-200b-3p, miR-223-3p, miR-375-3p, miR-4284 and let-7i-5p (catalog no.: YP00205702, YP00204230, YP00204291, YP00204688, YP00204099, YP00204080, YP00206071, YP00205986, YP00204362, YP02114835, YP00204394; product no.: 339306, Qiagen). qRT-PCR was performed using the SensiFAST™ SYBR^®^ Lo-ROX Kit (Bioline, London, UK) on the Quantstudio 6 Real-Time PCR System (Applied Biosystems, Foster City, CA, USA) and we used miR-16-5p as an endogenous control for data normalization. The relative expression levels of target genes were normalized against beta-actin using the 2-ΔCT method.

### 2.4. Statistical Analysis

We performed receiver operating characteristic (ROC) curve analysis and used area under the curve (AUC) values to evaluate the diagnostic power of the miRNA panel. For ROC analysis, we used a risk score calculated by a logistic regression model. The categorical factors were analyzed by Fisher’s exact test or chi-squared test and continuous factors were analyzed by Mann–Whitney U test. Spearman’s correlation coefficient was used to determine statistical dependence between variables. All statistical tests were two-sided and *p*-value < 0.05 was considered significant. All statistical analyses were performed using MedCalc statistical software Version 19.1 (Medcalc Software bvba, Ostend, Belgium), JMP software 14.0.0 (SAS Institute, Cary, NC, USA) and GraphPad Prism version 8.2.0 (GraphPad Software, San Diego, CA, USA). To identify the most robust miRNA panel for the diagnosis of UC patients, the adaptive LASSO model was applied to the qPCR data using JMP 14.0.0 (SAS Institute, Cary, NC, USA).

## 3. Results

### 3.1. Clinical Characteristics of Patients

A total of 60 patients with complete clinical data and with a sufficient amount of material from colonic tissue for further analysis were included in the final analysis. The basic demographic and clinical characteristics of patients are shown in Table 1.

As a controls, we used a group of 18 children without macroscopic and microscopic signs of IBD. The control group consisted of 12 girls (66.7%) and 6 boys (33.3%), with mean age of 14.6 years.

### 3.2. Expression of 10 Selected miRNAs

**Figure 1:**
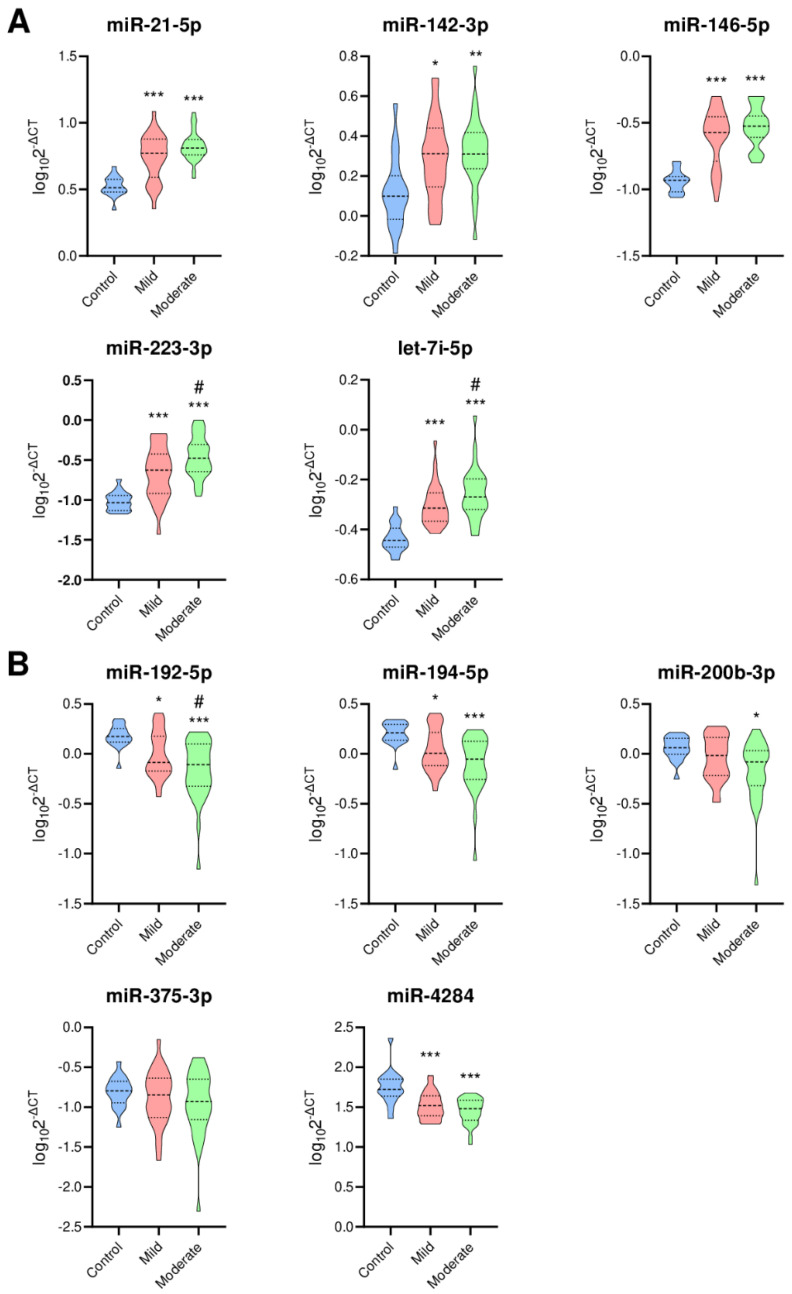
Expression of selected 10 miRNAs. (**A**) Upregulated miRNAs; (**B**) Downregulated miRNAs. Violin plots indicating expression of each miRNA. Blue, red and green violins indicate controls, mild UC patients and moderate UC patients, respectively. Thick dotted line, median; thin dotted line 25% and 75% quartiles. Y-axis is defined as log10(2-ΔCT). * *p* < 0.01, ** *p* < 0.001, *** *p* < 0.0001 versus control, # *p* < 0.05 versus mild UC patient.

Differences in expression of selected 10 miRNAs in patients with mild and moderate UC (based on PUCAI score) and healthy controls are depicted in Figure 1.

In the Supplementary materials, we provide the expression of 10 selected miRNAs (Supplementary Figure S1), ROC curves for the 10 individual miRNAs for distinguishing UC patients from controls (Supplementary Figure S2) and a model for diagnostic accuracy of 10-miRNA panel for identification of pediatric UC patients (Supplementary Figure S3).

### 3.3. Identification of Robust miRNA Panel for UC Diagnosis

**Figure 2:**
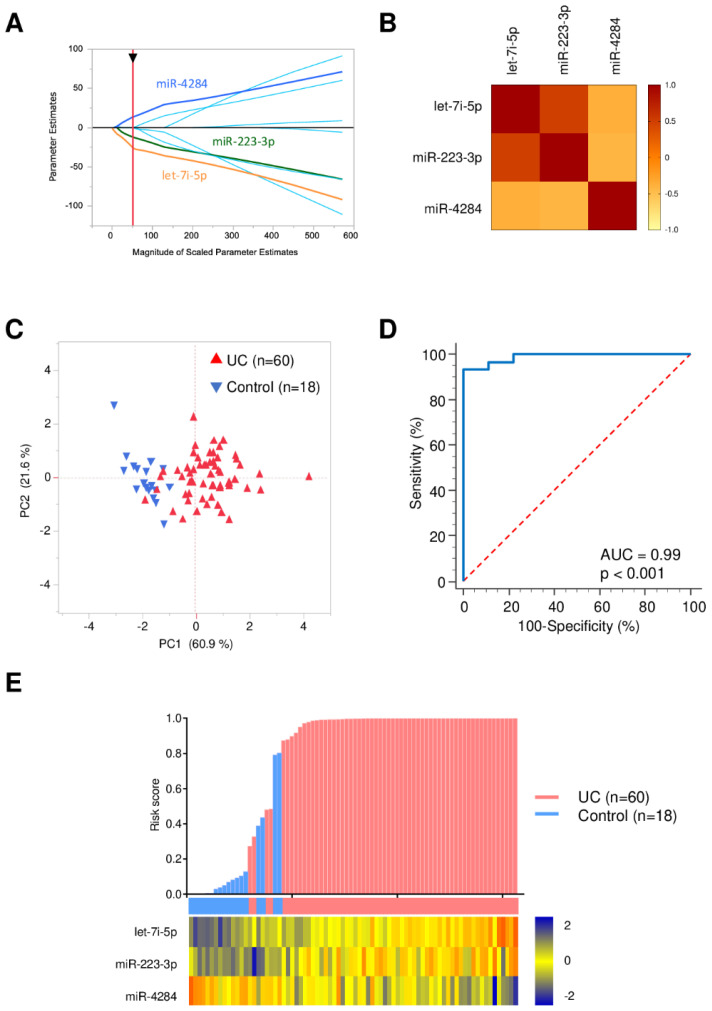
Diagnostic accuracy of 3-miRNA panel for identifying UC patients. (**A**) The adaptive LASSO model; (**B**) A correlation matrix displaying the Spearman’s rank correlation coefficient for each pair of three selected miRNAs; (**C**) Principal component analysis illustrating the good separation of UC-patient group and control group; (**D**) ROC curves for detecting UC patients using 3-miRNA panel; (**E**) A waterfall plot representing risk score of each patient. Red and blue columns indicate UC patients and controls, respectively. A heat map illustrating expression levels of the three candidate miRNAs expressed differentially between UC patients and controls.

To identify the most robust miRNA panel for the diagnosis of UC patients, the adaptive LASSO model was applied to the qPCR data using JMP 14.0.0 (SAS Institute, Cary, NC, USA). Three candidate miRNAs (let-7i-5p, miR-223-3p and miR-4284) were distinguished for accurate identification of pediatric UC patients (Figure 2). A risk score for detection of UC patients was calculated as follows: logit (risk score) = 37.84 + 38.89 × let-7i-5p + 5.46 × miR-223-3p − 10.65 × miR-4284.

### 3.4. Severity Stratification Panel

**Figure 3:**
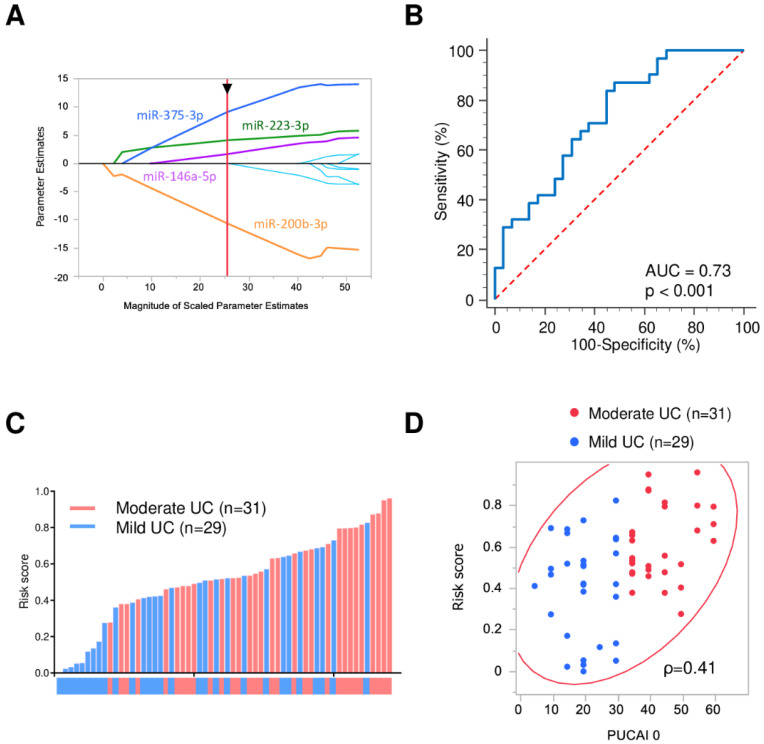
Diagnostic accuracy of 4-miRNA panel for identifying severity of UC patients. (**A**) Adaptive LASSO model; (**B**) ROC curves for detecting UC patients using 4-miRNA panel; (**C**) A waterfall plot representing risk score of each patient. Red and blue columns indicate moderate and mild UC patients, respectively; (**D**) A scatter plot showing the correlation of PUCAI with risk score. Red and blue circles indicate moderate and mild UC patients, respectively. ρ is the Spearman’s rank correlation coefficient.

In the quest to find the most robust miRNA panel for identifying UC severity, an adaptive LASSO model was applied to the qPCR data using JMP 14.0.0 (SAS Institute, Cary, NC, USA). Four candidate miRNAs (miR-375-3p, miR-146a-5p, miR-223-3p and miR-200b-3p) were prioritized for identifying severity of UC in pediatric patients (Figure 3).

We built a risk score for quantifying the severity of the UC patients as follows: logit (risk score) = 6.99 − 8.32 × miR-200b-3p + 2.45 × miR-223-3p + 5.61 × miR-375-3p + 2.50 × miR-146a-5p.

### 3.5. Early Relapse Prediction

**Figure 4:**
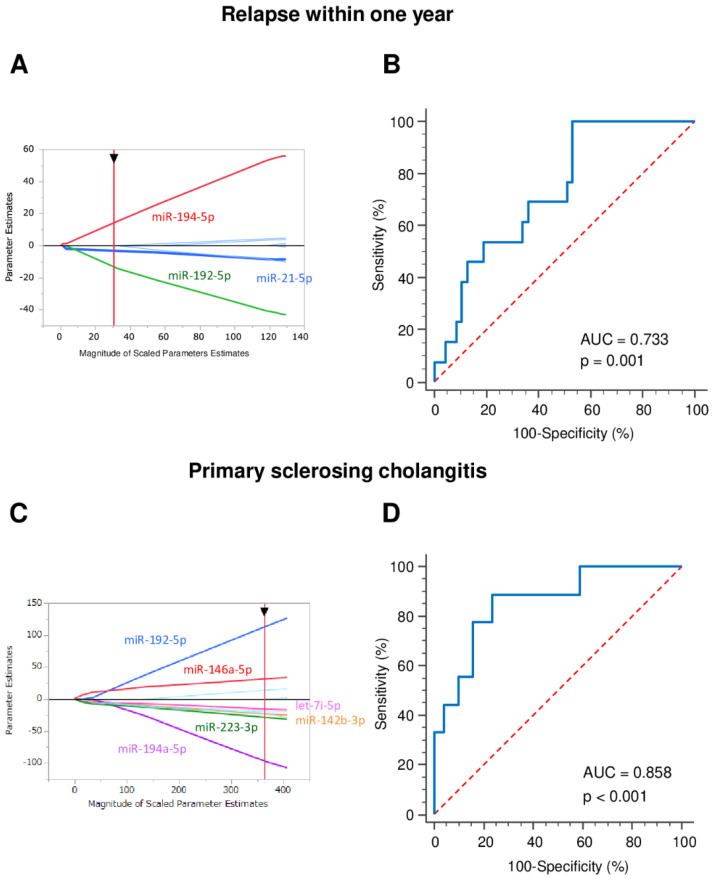
Diagnostic accuracy of miRNAs for identifying relapse within 1 year and primary sclerosing cholangitis. (**A**) Adaptive LASSO model; (**B**) ROC curves for detecting early relapse in UC patients using 3-miRNA panel. Logit (risk score) = 1.26 − 4.44 × miR-21-5p − 15.83 × miR-192-5p + 17.60 × miR-194-5p; (**C**) Adaptive LASSO model was applied to the qPCR data; (**D**) ROC curves for detecting primary sclerosing cholangitis in UC patients using 6-miRNA panel. Logit (risk score) = − 0.24 − 5.43 × miR-142-3p + 13.00 × miR-146a-5p + 14.59 × miR-192-5p − 19.57 × miR-194-5p − 5.23 × miR-223-3p − 14.42 × let-7i-5p.

Endeavoring to stratify patients according to their response to the standard therapeutic regime, we identified in the same manner three specific miRNAs (upregulated miR-21-5p and downregulated miR-192-5p and miR-194-5p) that appear to be associated with early disease relapse, where early relapse was assessed as a significant worsening of the patient’s condition during the first year of treatment (based on clinical score, laboratory results and elevated fecal calprotectin levels) (Figure 4).

### 3.6. Primary Sclerosing Cholangitis Identification

To determine the most robust miRNA panel for the identification of patients with UC and primary sclerosing cholangitis, an adaptive LASSO model was applied to the qPCR data using JMP 14.0.0 (SAS Institute, Cary, NC, USA). Four upregulated (miR-142-3p, miR-146a-5p, miR-223-3p and let-7i-5p) and two downregulated (miR-192-5p and miR-194-5p) miRNAs were identified (Figure 4).

## 4. Discussion

A knowledge of non-coding RNAs, mainly regarding miRNAs, has grown rapidly in recent years, mostly since the development of next-generation sequencing. Expression levels of miRNAs are closely related to many vital cellular processes and their regulation, as miRNAs act as fine-tuners of those processes. By complementary binding to the 3′-untranslated region of their target mRNA, miRNAs destabilize or activate the degradation of their target mRNA and thus preclude its translation. Being involved in a complex regulatory network consisting of not only other miRNAs but also of many other regulatory molecules, some miRNAs can regulate multiple pathways even as other miRNAs are simultaneously regulated by several pathways. Thus, a complex network exists and is involved also in IBD. Evidence on the involvement of miRNAs in the development of IBD is based mostly on studies performed on the adult population. Studies performed on cohorts of pediatric patients are rather sparse and of limited reliability, as most of the studies work with several dozen patients at most. Despite all the limitations, the results are mutually confirming [12], at least in the cases of several miRNAs, namely miR-21, miR-146a and miR-142-3p [18,19,20].

Most of the previously published studies were focused on differentiating IBD cases from healthy controls, thus identifying so-called diagnostic biomarkers. We tested, also, the ability of chosen miRNAs to differentiate UC cases from healthy patients, which was feasible using a combination of let-7i, miR-223 and miR-4284. There exist, however, many more specific clinical challenges, such as responsiveness to therapy, prediction of relapse, differentiation of IBD subtypes and preventing overtreatment. We therefore focused our effort also on other biomarker aspects and successfully identified a combination of miR-375, miR-146a, miR-223 and miR-200b that was able to identify patients with a more severe course of the disease. Moreover, we identified that the upregulation of miR-21 and downregulation of miR-192 and miR-194 are indicative of early relapse after treatment and more precisely relapse within 1 year after administering treatment. In order to find a biomarker discerning patients with IBD associated with PSC (PSC-IBD), we showed for the first time that a combination of 3 upregulated miRNAs (miR-142-3p, miR-146a-5p and miR-223-3p) and 3 downregulated miRNAs (miR-192-5p, miR-194-5p and miR-375-3p) is typical for PSC-IBD patients.

Although IBD’s exact pathogenesis remains unclear, its inflammatory nature is evident also from miRNAs that are repeatedly identified as deregulated in IBD patients of either subtype [12]. Potential involvement of those miRNAs can be traced to the most essential pathways and agents of systemic inflammatory response, such as TLR/NF-κB, TNF-α and other pro- and anti-inflammatory factors, either as their regulators (as in the case of miR-375,3 miR-223 [21,22], let-7i [23], miR-200b and the entire miR-200 family [24,25] and miR-146a [26]) or as the molecules regulated by such pro- and anti-inflammatory agents, such as miR-142 [27,28]. Closely related to inflammation seems to be autophagy, a natural process of removing certain cellular components. Among others, it is involved in maintaining homeostasis and survival of such inflammatory cells as lymphocytes, neutrophils and macrophages [29] miR-223, miR-146a [26,30], miR-142 [28], miR-192 [31] and miR-194 [26] are all involved in regulation of autophagy and thus, in inflammation through NOD2, ATG16L1 and mTOR pathways [29]. A specific spot is held by miR-21, notoriously known as a potent inflammatory switch and a key regulator of both pro- and anti-inflammatory factors, which acts as a “molecular rheostat” [32]. Moreover, persistent inflammation is a prerequisite for carcinogenesis and consistent with this are findings of miRNAs upregulated in IBD and their involvement in the development of cancer, such as miR-21 through PCDC4 downregulation [33] and miR-4284 through the CXCL5 pathway and regulation of chemotaxis and proliferation [34,35,36].

In our study, we have successfully validated results and confirmed biomarker potential in several miRNAs identified in studies of similar design. Moreover, to the best of our knowledge, we have evaluated for the first time in a Central European representative population of children with ulcerative pancolitis expression of selected miRNAs and their association with key clinical questions. Our study cohort is relatively homogenous, consisting exclusively of well-described pancolitic disease patients, thus avoiding any inconsistency stemming from an uneven range of the disease among patients. The results demonstrate that the specific combinations of expressed miRNAs in colonic mucosa are associated with important clinical parameters for more precise diagnosis, prediction of prognosis and clinical outcome. The uniqueness of our study lies in an unusually large study cohort. No previous study has been carried out on more than 20 patients with UC, and therefore, the reliability of the information has been somewhat limited. Independent validation of the results on a cohort including as many as 78 pediatric patients has never been published to date. Moreover, we have identified miRNAs and their combinations that not only can differentiate patients from controls but also patients with early relapse of the disease. This is of great diagnostic and prognostic importance. Due to our stratification of patients is based solely on the PUCAI score, however and that score itself has some diagnostic limitations, our results should be verified on a cohort described more precisely by other clinical and endoscopic (Mayo score) parameters.

In addition, unique is the identification of patients with PSC-IBD. Although typical for middle-aged males, this condition also affects children. In the majority of the childhood cases, it is associated with IBD, typically with UC, while association with CD is less common [5,37]. The course of PSC-IBD and IBD alone is similar, but colon dysplasia may occur in patients with PSC-IBD and this predisposes them to the development of colon cancer later in life. Those patients are therefore at greater risk and need more frequent follow-up [4,38]. Early identification of PSC-IBD patients could lead to more effective care.

As current therapeutic options do not allow for early identification of patients who would not profit from a standard therapy either for non-responsiveness or for other causes, identification of potential biomarkers is of utmost interest for physicians. It should be noted, though, that the miRNAs validated in our study and successfully confirmed as deregulated are involved in inflammation as such, and therefore, do not reflect specific IBD changes other than overall inflammation of the gut. Moreover, this study validates previously published results. The main limitation of our study is a lack of the CD patients cohort disabling determination of the specificity of tested biomarkers.

While this study undeniably brings value to the existing knowledge, independent validation in combination with profiling that uses such currently available high-throughput techniques as next-generation sequencing would be even more significant.

## 5. Conclusions

In conclusion, we independently verified the biomarker potential of miRNAs feasible for assessing detection and prognosis and discovered that their combinations could distinguish not only UC patients from controls without signs of IBD but even more specific conditions such as disease severity, early relapse and even association with PSC. A larger cohort of patients with not only UC but also CD and a study based on expression profiling using high-throughput platforms and independent validation is greatly needed in this field.

## Figures and Tables

**Table 1:** Demografic and clinical characteristics of UC patients.

Severity of the Disease (PUCAI Score)	Mild	Moderate	Total
	29 (48.3%)	31 (51.7%)	60 (100%)
Sex, N (%)			
Male	13 (44.8%)	13 (41.9%)	26
Female	16 (55.2%)	18 (58.1%)	34
Mean age at diagnosis (years)	13.7	13.9	13.7
Mean PUCAI:			
At diagnosis	20.7	43.7	32.3
After 3 months	2.2	5.0	3.7
Treatment, N (%)			
Mesalazine	29 (100%)	31 (100%)	60 (100%)
Corticosteroids	18 (62%)	25 (80.6%)	43 (71.7%)
Response rate to initial treatment, N (%)	26 (89.7%)	25 (80.6%)	51 (85%)
Early relapse, N (%)	6 (20.7%)	7 (22.6%)	13 (21.7%)
Primary sclerosing cholangitis, N (%)	6 (20.7%)	3 (9.7%)	9 (15%)

## Data Availability

The data underlying this article cannot be shared publicly due to ethical reasons. The data will be shared on reasonable request to the corresponding author.

## Supplementary Materials

The following are available online at https://www.mdpi.com/2077-0383/10/6/1325/s1, Supplementary Figure S1: Expression of 10 selected miRNAs, Supplementary Figure S2: Receiver operating characteristic (ROC) curves for 10 individual miRNAs for distinguishing UC patients from controls, Supplementary Figure S3: Diagnostic accuracy of 10-miRNA panel for identifying UC patients.

## References

[1] Sartor R.B. (2006). Mechanisms of disease: Pathogenesis of Crohn’s disease and ulcerative colitis. Nat. Clin. Pract. Gastroenterol. Hepatol..

[2] Cao B., Zhou X., Ma J., Zhou W., Yang W., Fan D., Hong L. (2017). Role of MiRNAs in inflammatory bowel disease. Dig. Dis. Sci..

[3] Wu C.P., Bi Y.J., Liu D.-M., Wang L.-Y. (2019). Hsa-miR-375 promotes the progression of inflammatory bowel disease by upregulating TLR4. Eur. Rev. Med. Pharmacol. Sci..

[4] Rosen M.J., Karns R., Vallance J.E., Bezold R., Waddell A., Collins M.H., Haberman Y., Minar P., Baldassano R.N., Hyams J.S. (2017). Mucosal expression of type 2 and Type 17 immune response genes distinguishes ulcerative colitis from colon-only Crohn’s disease in treatment-naive pediatric patients. Gastroenterology.

[5] Laborda T.J., Jensen M.K., Kavan M., Deneau M. (2019). Treatment of primary sclerosing cholangitis in children. World J. Hepatol..

[6] Jabandziev P., Pinkasova T., Kunovsky L., Papez J., Jouza M., Karlinova B., Novackova M., Urik M., Aulicka S., Slaby O. (2020). Regional incidence of inflammatory bowel disease in a Czech pediatric population: 16 years of experience (2002–2017). J. Pediatr. Gastroenterol. Nutr..

[7] Sýkora J., Pomahačová R., Kreslová M., Cvalínová D., Štych P., Schwarz J. (2018). Current global trends in the incidence of pediatric-onset inflammatory bowel disease. World J. Gastroenterol..

[8] Kelsen J., Baldassano R.N. (2008). Inflammatory bowel disease: The difference between children and adults. Inflamm. Bowel Dis..

[9] Turunen P., Ashorn M., Auvinen A., Iltanen S., Huhtala H., Kolho K.-L. (2009). Long-term health outcomes in pediatric inflammatory bowel disease: A population-based study. Inflamm. Bowel Dis..

[10] Guariso G., Gasparetto M. (2017). Treating children with inflammatory bowel disease: Current and new perspectives. World J. Gastroenterol..

[11] Naviglio S., Lacorte D., Lucafò M., Cifù A., Favretto D., Cuzzoni E., Silvestri T., Mucelli M.P., Radillo O., Decorti G. (2019). Causes of treatment failure in children with inflammatory bowel disease treated with infliximab: A pharmacokinetic study. J. Pediatr. Gastroenterol. Nutr..

[12] Jabandziev P., Bohosova J., Pinkasova T., Kunovsky L., Slaby O., Goel A. (2020). The emerging role of noncoding RNAs in pediatric inflammatory bowel disease. Inflamm. Bowel Dis..

[13] IBD Working Group of the European Society for Paediatric Gastroenterology HpaN (2005). Inflammatory bowel disease in children and adolescents: Recommendations for diagnosis—The Porto criteria. J. Pediatr. Gastroenterol. Nutr..

[14] Levine A., Koletzko S., Turner D., Escher J.C., Cucchiara S., de Ridder L., Kolho K.-L., Veres G., Russell R.K., Paerregaard A. (2014). ESPGHAN revised porto criteria for the diagnosis of inflammatory bowel disease in children and adolescents. J. Pediatr. Gastroenterol. Nutr..

[15] Levine A., Griffiths A., Markowitz J., Wilson D.C., Turner D., Russell R.K., Fell J., Ruemmele F.M., Walters T., Sherlock M. (2011). Pediatric modification of the Montreal classification for inflammatory bowel disease: The Paris classification. Inflamm. Bowel Dis..

[16] Turner D., Hyams J., Markowitz J., Lerer T., Mack D.R., Evans J., Pfefferkorn M., Rosh J., Kay M., Crandall W. (2009). Appraisal of the pediatric ulcerative colitis activity index (PUCAI). Inflamm. Bowel Dis..

[17] Bronský J., Beránková K., Černá Z., Čopová I., Durilová M., Hradský O., Karásková E., Mitrová K., Nevoral J., Poš L. (2017). Czech Working Group for Paediatric Gastroenterology and Nutrition guidelines for diagnostics and treatment of inflammatory bowel diseases in children—1st edition update. Gastroenterol. Hepatol..

[18] Zahm A.M., Hand N.J., Tsoucas D.M., Le Guen C.L., Baldassano R.N., Friedman J.R. (2014). Rectal microRNAs are perturbed in pediatric inflammatory bowel disease of the colon. J. Crohns Colitis.

[19] Béres N.J., Szabó D., Kocsis D., Szűcs D., Kiss Z., Müller K.E., Lendvai G.A., Kiss A., Arató A., Sziksz E. (2016). Role of altered expression of miR-146a, miR-155, and miR-122 in pediatric patients with inflammatory bowel disease. Inflamm. Bowel Dis..

[20] Béres N.J., Kiss Z., Sztupinszki Z., Lendvai G., Arató A., Sziksz E., Vannay Á., Szabó A.J., Müller K.E., Cseh Á. (2017). Altered mucosal expression of microRNAs in pediatric patients with inflammatory bowel disease. Dig. Liver Dis..

[21] Leon-Cabrera S., Vázquez-Sandoval A., Molina-Guzman E., Delgado-Ramirez Y., Delgado-Buenrostro N.L., Callejas B.E., Chirino Y.I., Pérez-Plasencia C., Rodríguez-Sosa M., Olguín J.E. (2018). Deficiency in STAT1 signaling predisposes gut inflammation and prompts colorectal cancer development. Cancers.

[22] Giles E.M., Sanders T.J., McCarthy N.E., Lung J., Pathak M., Macdonald T.T., Lindsay J.O., Stagg A.J. (2017). Regulation of human intestinal T-cell responses by type 1 interferon-STAT1 signaling is disrupted in inflammatory bowel disease. Mucosal Immunol..

[23] Giroud M., Karbiener M., Pisani D.F., Ghandour R.A., Beranger G.E., Niemi T., Taittonen M., Nuutila P., Virtanen K.A., Langin D. (2016). Let-7i-5p represses brite adipocyte function in mice and humans. Sci. Rep..

[24] Chen Y., Zhang L. (2017). Members of the microRNA-200 family are promising therapeutic targets in cancer. Exp. Ther. Med..

[25] Lewis A., Felice C., Kumagai T., Lai C., Singh K., Jeffery R.R., Feakins R., Giannoulatou E., Armuzzi A., Jawad N. (2017). The miR-200 family is increased in dysplastic lesions in ulcerative colitis patients. PLoS ONE.

[26] Wang S., Huang Y., Zhou C., Wu H., Zhao J., Wu L., Zhao M., Zhang F., Liu H. (2018). The role of autophagy and related MicroRNAs in inflammatory bowel disease. Gastroenterol. Res. Pract..

[27] Duijvis N.W., Moerland P.D., Kunne C., Slaman M.M.W., Van Dooren F.H., Vogels E.W., De Jonge W.J., Meijer S.L., Fluiter K., Velde A.A.T. (2017). Inhibition of miR-142-5P ameliorates disease in mouse models of experimental colitis. PLoS ONE.

[28] Lu Y., Gao J., Zhang S., Gu J., Lu H., Xia Y., Zhu Q., Qian X., Zhang F., Zhang C. (2018). miR-142-3p regulates autophagy by targeting ATG16L1 in thymic-derived regulatory T cell (tTreg). Cell Death Dis..

[29] Qian M., Fang X., Wang X. (2017). Autophagy and inflammation. Clin. Transl. Med..

[30] Sonkoly E., Ståhle M., Pivarcsi A. (2008). MicroRNAs and immunity: Novel players in the regulation of normal immune function and inflammation. Semin. Cancer Biol..

[31] Lin J., Zhang X., Zhao Z., Welker N.C., Li Y., Liu Y., Bronner M.B. (2016). Novel MicroRNA signature to differentiate ulcerative colitis from Crohn disease: A genome-wide study using next generation sequencing. Microrna.

[32] Sheedy F.J. (2015). Turning 21: Induction of miR-21 as a key switch in the inflammatory response. Front. Immunol..

[33] Ludwig K., Fassan M., Mescoli C., Pizzi M., Balistreri M., Albertoni L., Pucciarelli S., Scarpa M., Sturniolo G.C., Angriman I. (2013). PDCD4/miR-21 dysregulation in inflammatory bowel disease-associated carcinogenesis. Virchows Arch. Pathol. Anat. Physiol. Klin. Med..

[34] Persson T., Monsef N., Andersson P., Bjartell A., Malm J., Calafat J., Egesten A. (2003). Expression of the neutrophil-activating CXC chemokine ENA-78/CXCL5 by human eosinophils. Clin. Exp. Allergy.

[35] Koukos G., Polytarchou C., Kaplan J.L., Oikonomopoulos A., Ziring D., Hommes D.W., Wahed R., Kokkotou E., Pothoulakis C., Winter H.S. (2015). A microRNA signature in pediatric ulcerative colitis: Deregulation of the miR-4284/CXCL5 pathway in the intestinal epithelium. Inflamm. Bowel Dis..

[36] Zhang R., Liu Q., Peng J., Wang M., Li T., Liu J., Cui M., Zhang X., Gao X., Liao Q. (2020). CXCL5 overexpression predicts a poor prognosis in pancreatic ductal adenocarcinoma and is correlated with immune cell infiltration. J. Cancer.

[37] Tenca A., Jaakkola T., Färkkilä M., Arola J., Kolho K.-L. (2019). Impact of paediatric onset primary sclerosing cholangitis on clinical course and outcome of inflammatory bowel disease: A case-control population-based study in Finland. Scand. J. Gastroenterol..

[38] Dyson J.K., Beuers U., Jones D.E.J., Lohse A.W., Hudson M. (2018). Primary sclerosing cholangitis. Lancet.

