# Supplementary material for: MicroRNAs in Colon Tissue of Pediatric Ulcerative Pancolitis Patients Allow Detection and Prognostic Stratification
Source: J Clin Med. 2021 Mar 23;10(6):1325. doi: 10.3390/jcm10061325 (PMC8005023; doi:10.3390/jcm10061325)
Supplement: Supplementary file 1 [file jcm-10-01325-s001.pdf]

Supplementary Figure 1

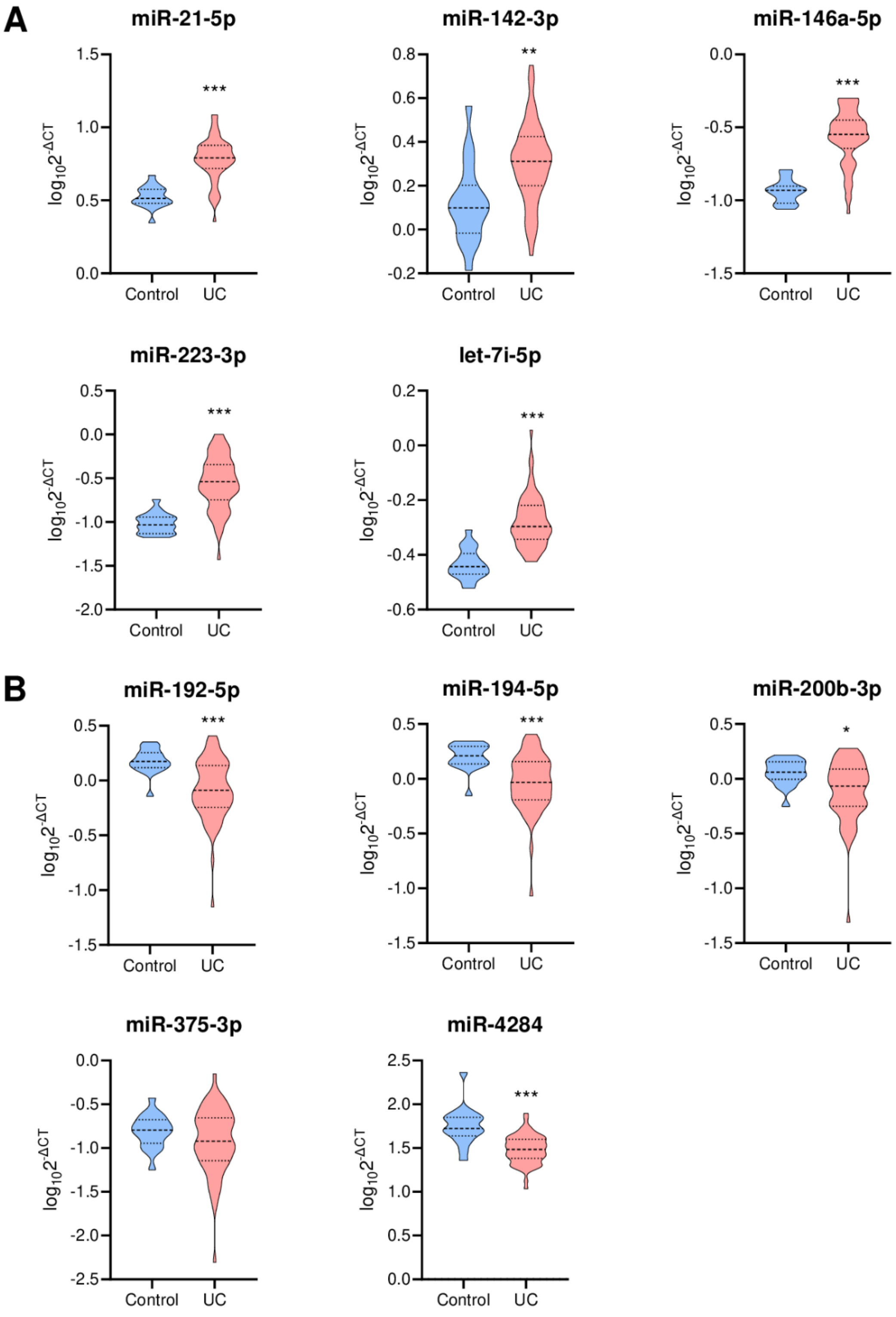

Supplementary Figure S1. Expression of 10 selected miRNAs.

Violin plots indicating expression of each miRNA. Blue and red violins indicate controls and ulcerative colitis (UC) patients, respectively. Thick dotted line, median; thin dotted line 25% and 75% quartiles. Y-axis is defined as $\log_{10}(2^{-\Delta CT})$. *$p < 0.01$, **$p < 0.001$, ***$p < 0.0001$ versus control.

Supplementary Figure 2

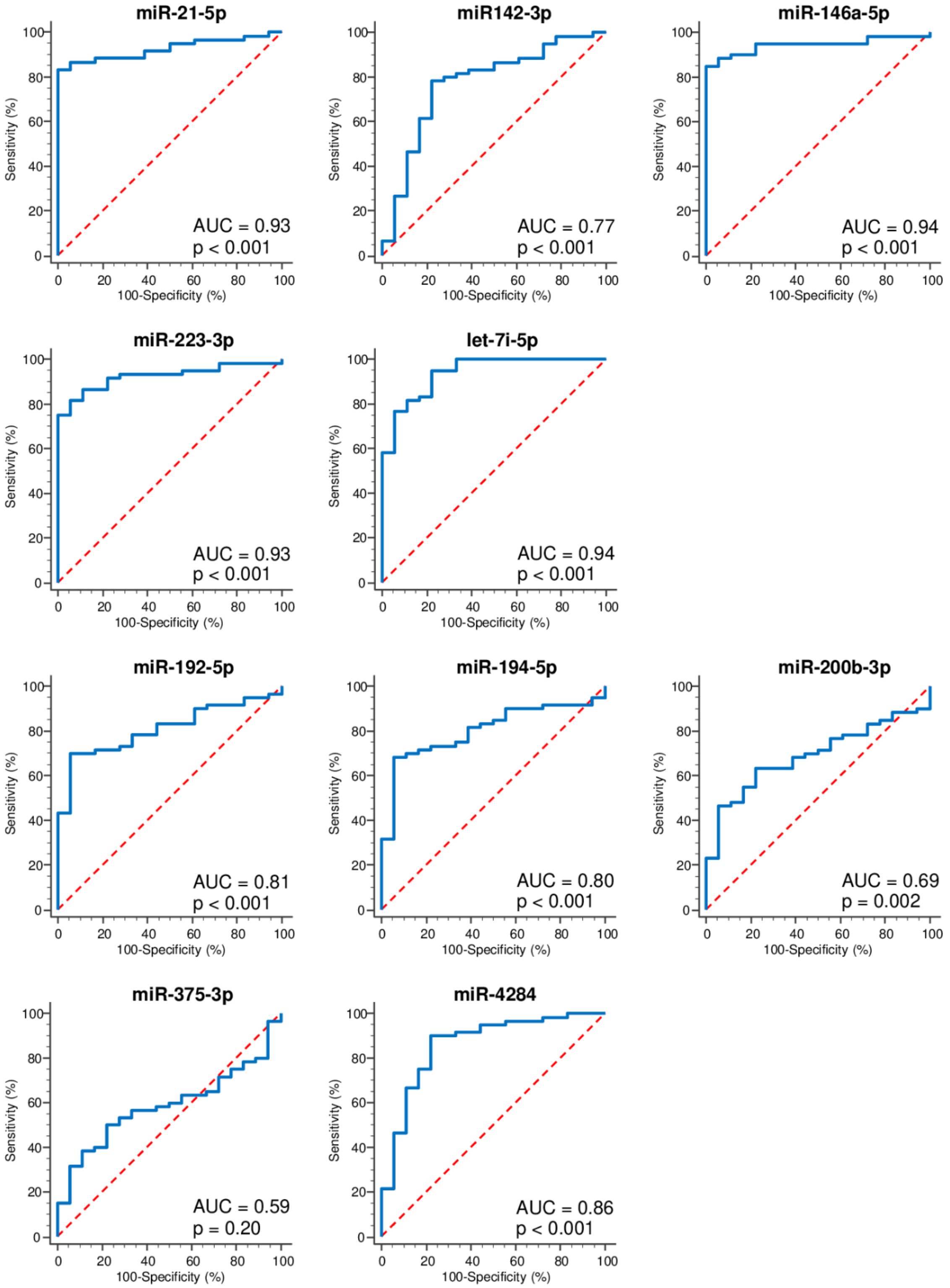

Supplementary Figure S2. Receiver operating characteristic (ROC) curves for 10 individual miRNAs for distinguishing UC patients from controls.

Supplementary Figure 3

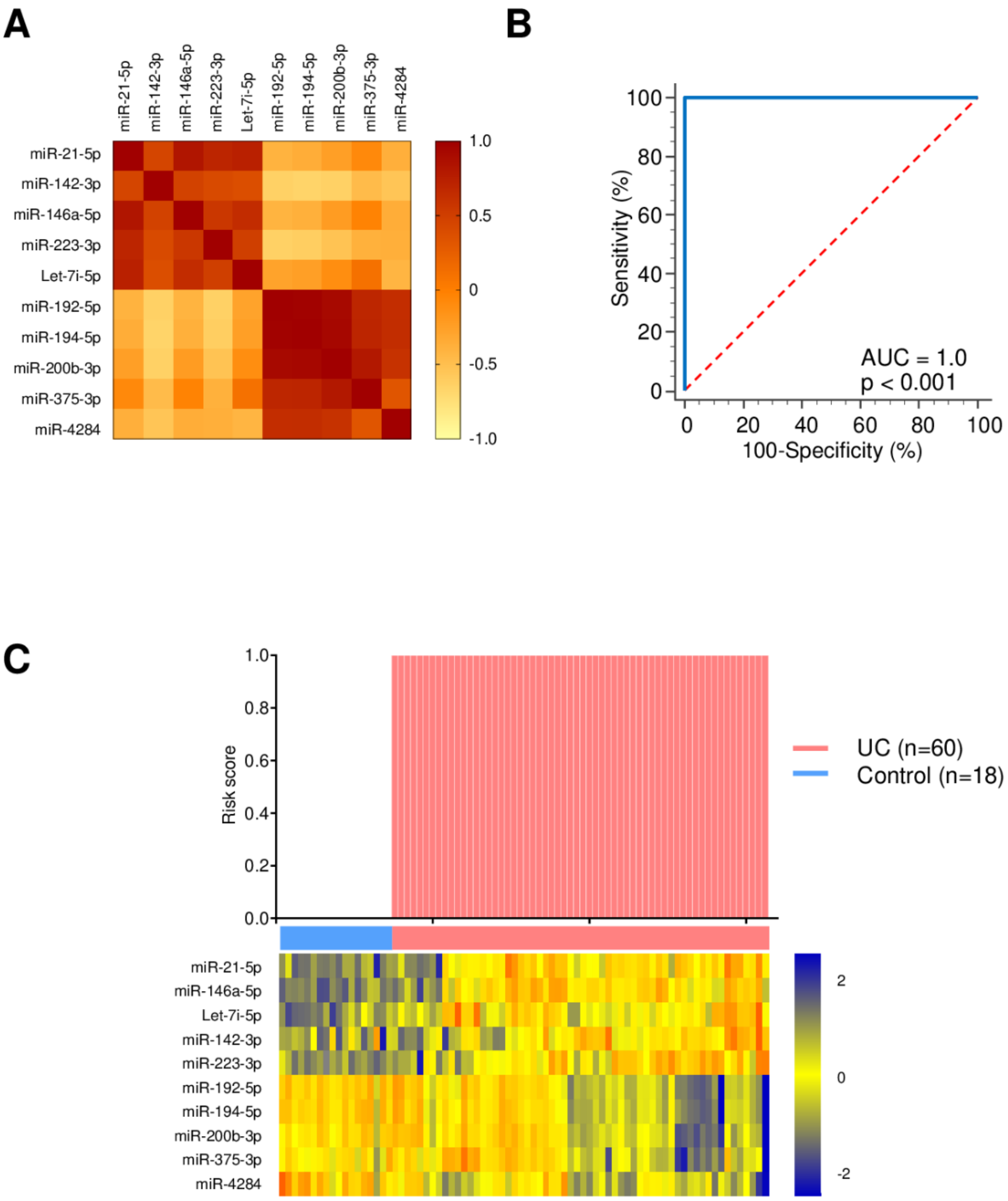

Supplementary Figure S3. Diagnostic accuracy of 10-miRNA panel for identifying UC patients.

(A) A correlation matrix displaying the Spearman's rank correlation coefficient for each pair of 10 miRNAs. (B) Receiver operating characteristic (ROC) curves for detecting UC patients using 10-miRNA panel. Logit (risk score) = 1857.24 + 1471.51 × let-7i-5p – 83.19 × miR-142-3p + 170.29 × miR-146a-5p + 701.10 × miR-

192-5p – 528.76 × miR-194-5p + 471.34 × miR-200b-3p – 144.90 × miR-21-5p + 398.23 × miR-223-3p – 344.13 × miR-375-3p – 594.95 × miR-4284. (C) A waterfall plot representing risk score of each patient. Red and blue columns indicate UC patients and controls, respectively. A heat map illustrating the expression levels of 10 miRNAs expressed differentially between UC patients and controls.
